# Anti-Inflammatory Influences of Cystic Fibrosis Transmembrane Conductance Regulator Drugs on Lung Inflammation in Cystic Fibrosis

**DOI:** 10.3390/ijms22147606

**Published:** 2021-07-16

**Authors:** Kiera H. Harwood, Rachel M. McQuade, Andrew Jarnicki, Elena K. Schneider-Futschik

**Affiliations:** 1 Department of Biochemistry & Pharmacology, Faculty of Medicine, Dentistry and Health Sciences, School of Biomedical Sciences, The University of Melbourne, Parkville, VIC 3010, Australia; kharwood@student.unimelb.edu.au; 2 Gut-Axis Injury and Repair Laboratory, Department of Medicine Western Health, Melbourne University, Melbourne, VIC 3021, Australia; rachel.mcquade@unimelb.edu.au; 3 The Florey Institute of Neuroscience and Mental Health, Parkville, VIC 3010, Australia; 4 Lung Disease Research Laboratory, Department of Biochemistry & Pharmacology, Melbourne University, Melbourne, VIC 3021, Australia

**Keywords:** cystic fibrosis, ivacaftor, lumacaftor, CFTR modulator, inflammation, lung inflammation, anti-inflammatory treatment

## Abstract

Cystic fibrosis (CF) is caused by a defect in the cystic fibrosis transmembrane conductance regulator protein (CFTR) which instigates a myriad of respiratory complications including increased vulnerability to lung infections and lung inflammation. The extensive influx of pro-inflammatory cells and production of mediators into the CF lung leading to lung tissue damage and increased susceptibility to microbial infections, creates a highly inflammatory environment. The CF inflammation is particularly driven by neutrophil infiltration, through the IL-23/17 pathway, and function, through NE, NETosis, and NLRP3-inflammasome formation. Better understanding of these pathways may uncover untapped therapeutic targets, potentially reducing disease burden experienced by CF patients. This review outlines the dysregulated lung inflammatory response in CF, explores the current understanding of CFTR modulators on lung inflammation, and provides context for their potential use as therapeutics for CF. Finally, we discuss the determinants that need to be taken into consideration to understand the exaggerated inflammatory response in the CF lung.

## 1. Introduction

**Figure 1:**
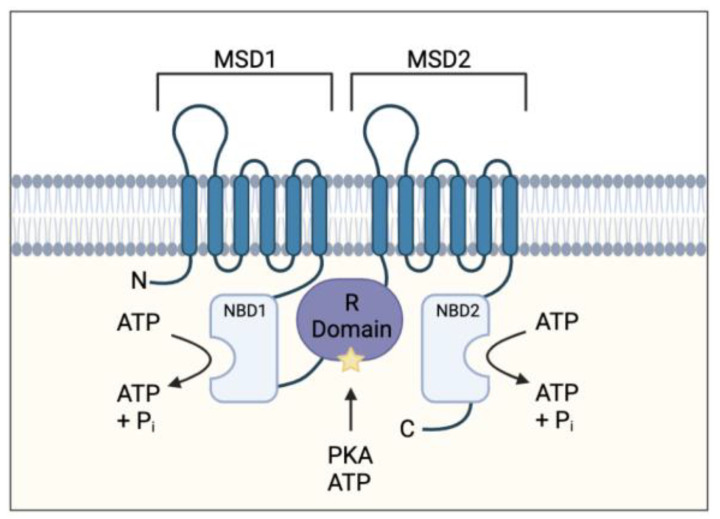
Cystic fibrosis transmembrane conductance regulator (CFTR) protein structure. Protein consists of two membrane-spanning domains (MSD), typically comprising of six transmembrane regions each which form the channel pore allowing chloride and bicarbonate transport; two nucleotide binding domains (NBD) which bind and hydrolyse ATP allowing for the channel to open; a single regulatory (R) domain, containing numerous charged amino acid and phosphorylation sites (star representing a phosphorylation site). The channel opens when R domain is phosphorylated, and NBDs are ATP-bound (adapted from Sheppard et al Physiol Rev. 1999 January 79 (1 Suppl): S23–S45).

Cystic fibrosis (CF) is an inherited, life-limiting disease most common in individuals of northern European descent affecting approximately 1 in 3000 births [1]. The disease is caused by mutations in the cystic fibro sis transmembrane conductor regulator (CFTR) gene. More than 2000 mutations have been identified and classified into 6 mutation classes depending on the type and severity of defective CFTR protein synthesis and function [2]. The CFTR gene codes for an ATP-binding cassette transporter class C ATPase protein present throughout the body, which allows chloride and bicarbonate ion transport at the apical membrane of epithelial cells [1]. It also plays a role in inhibiting sodium transport at these epithelial sites (Figure 1) [3]. 

**Figure 2:**
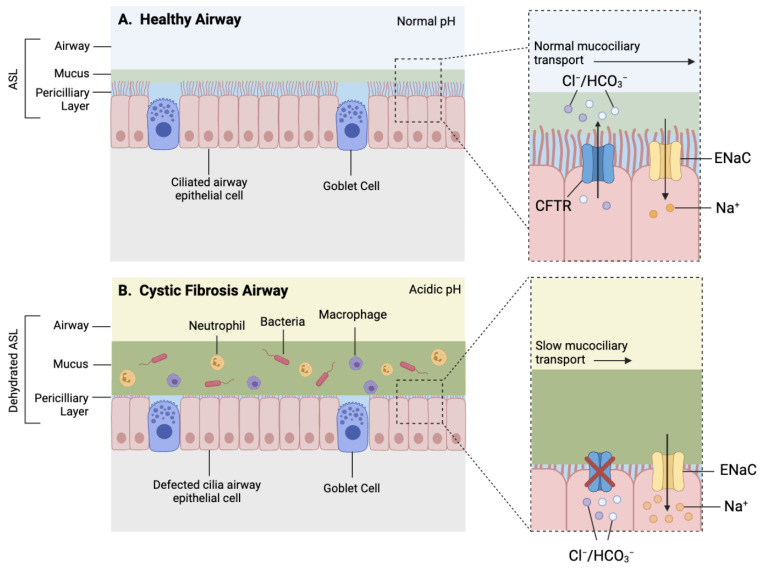
Simplified schematic of a healthy and CF airway. (**A**) Normal functioning CFTR channel in healthy airway enables efficient Chloride (Cl^−^) and bicarbonate (HCO_3_^−^) transport, as well as regulated sodium (Na^+^) reabsorption through epithelial sodium channel (ENaC), resulting in homeostatic airway microenvironment, with appropriate pH, airway surface liquid (ASL) hydration and mucus viscosity, promoting efficient mucociliary transport. (**B**) Dysfunctional CFTR protein in cystic fibrosis airway results in inhibited chloride and bicarbonate ion transport and increased sodium reabsorption which leads to reduced volume of periciliary layer, increased viscosity of mucus layer and acidic airway microenvironment contributing to mucostasis and inefficient mucociliary transport. Increased mucus viscosity promotes mucus plugging in submucosal gland, further preventing mucus clearance (not shown). Reduced mucus clearance and acidic airway pH contribute to a pro-inflammatory airway microenvironment consisting of increased influx of inflammatory cells and accumulation of bacteria within mucus, accelerating pathogenesis.

CFTR function influences the viscosity and pH of mucosal surfaces within several organs such as the lungs, pancreas, and intestines; hence, it is often associated with a vast range of comorbidities [4]; however, respiratory disease remains the key driver of morbidity and mortality within CF patients [5,6]. CF lung pathophysiology is characterised by depleted airway surface liquid (ASL), altered mucosal properties, decreased airway pH, and a dysregulated immune response, leading to irreversible bronchiectasis, recurring lung infections, and progressive respiratory decline [3,4] (Figure 2). 

The recent development of CFTR modulators, namely ivacaftor, lumacaftor, tezacaftor and elexacaftor, has reinvigorated therapeutic potential of resolving CFTR dysfunction directly as previous treatment options were only targeting secondary manifestations and symptoms [7]. This review aims to summarise the relationship between CF and lung inflammation, to explore current literature on the anti-inflammatory effects of CFTR modulators, and to propose future research avenues, all aimed to refine our understanding of this life-threatening disease and improve patient outcome.

## 2. CF-Induced Lung Inflammation 

### 2.1. Persistent Lung Infections

CFTR dysfunction instigates a myriad of respiratory complications including increased vulnerability to lung infections. *Staphylococcus aureus* (*S. aureus*) is the most prominent pathogen detected in children with CF [8]. Infection with *S. aureus* was found to be associated with a decline in lung function, as well as enhancing other pro-inflammatory mediators in the bronchoalveolar fluid (BALF), further exacerbating inflammation [9]. Once these CF children reach adulthood, approximately 80% are chronically infected with *Pseudomonas aeruginosa* (*P. aeruginosa*) [10]. Chronic colonisation of *P. aeruginosa* is extremely challenging to treat and is directly correlated to progressive respiratory decline and an increased mortality [10,11].

Insufficient bacterial clearance may be due to the inherent CFTR defects present in CF. Defective CFTR inhibits anion transport within the airways, intrinsically altering the state of its microenvironment disrupting both physical and chemical airway defence mechanisms [12]. CFTR dysfunction directly hampers the protective mechanism of mucociliary clearance by increasing ASL viscosity and promoting mucus tethering, leading to decreased mucociliary clearance [12,13,14]. This further prevents the elimination of bacteria accumulated in the mucus, increasing the likelihood of infection and subsequent inflammatory reactions [12]. CFTR dysfunction also inhibits the transport of bicarbonate ions through the airway lumen, altering the pH of the airway microenvironment. Changes in airway pH suppress the activity of antimicrobial peptides, altering the hosts innate immune response [15]. This theory has been reinforced by the observation that a reduction in antimicrobial activity occurs when the pH of the ASL in pigs was decreased, and an increased antimicrobial activity when the pH was increased [16]. Persistent lung infection heavily influences the infiltration and activation of proinflammatory cells resulting in progressive lung inflammation and defective barriers against subsequent infections.

### 2.2. Dysregulated Lung Inflammatory Response in CF

**Figure 3:**
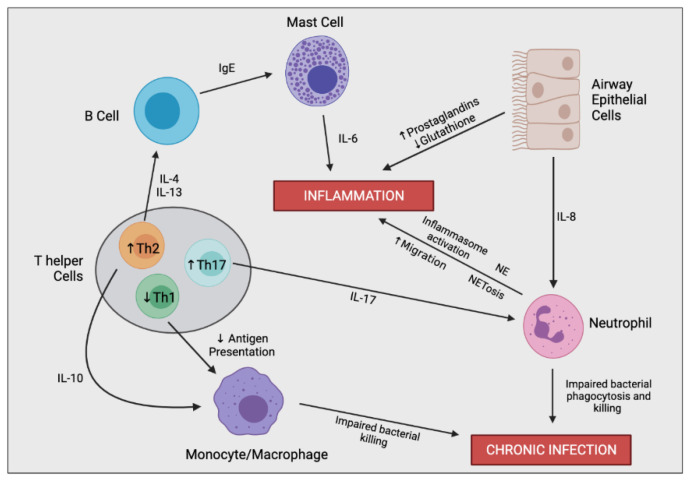
Simplified schematic outlining the complex immune cell interaction involved in producing the hyperinflammatory response and chronic infection in CF. In the CF airway, airway epithelial cells (AECs) secrete increased levels of IL-8, resulting in enhanced neutrophil migration. Perturbed secretion of prostaglandins and glutathione from AECs promotes inflammation. Neutrophils are the predominant driver of airway inflammation through multiple mechanisms including increased inflammasome activation, increased secretion of neutrophil elastase (NE), and a bias towards neutrophil extracellular trap (NET)-mediated cell death (NETosis). Neutrophils also possess impaired bacterial phagocytosis and killing leading to insufficient bacterial clearance and chronic infection. CF-macrophages also secrete increased levels of IL-8 and other proinflammatory mediators (not shown), promoting neutrophil infiltration and further inflammation directly. CF-macrophages also undergo impaired bacterial killing encouraging persistent infections. T helper cells in CF are skewed towards Th2 and Th17 differentiation rather than a Th1 phenotype. Th17 cells secrete IL-17, promoting neutrophil infiltration, as well as other proinflammatory cytokines which contribute to the hyperinflammatory response (not shown). Th2 cells stimulate a pro-allergic response involving increased secretion of IL-4 and IL-13 leading to IgE production. Th2 cells also secrete IL-10, dampening the expression of co-stimulatory molecules on macrophages causing decreased antigen presentation and impaired bacterial clearance.

The excessive inflammatory cascade begins with the first airway infection, typically at an early age, and persists throughout the lifetime [17]. Sustained inflammation is predominantly driven by an imbalance in pro-inflammatory and pro-resolving mediators. Reduction in pro-resolving mediators promotes further immune cell recruitment into the airways, leading to continuous airway damage and remodelling [18]. This cycle is characteristic for CF and drives the inevitable decrease in lung function. The relationship between inflammation and the decline in lung function involves a complex interplay between multiple different cell types (Figure 3).

#### 2.2.1. Epithelial Cells

**Figure 4:**
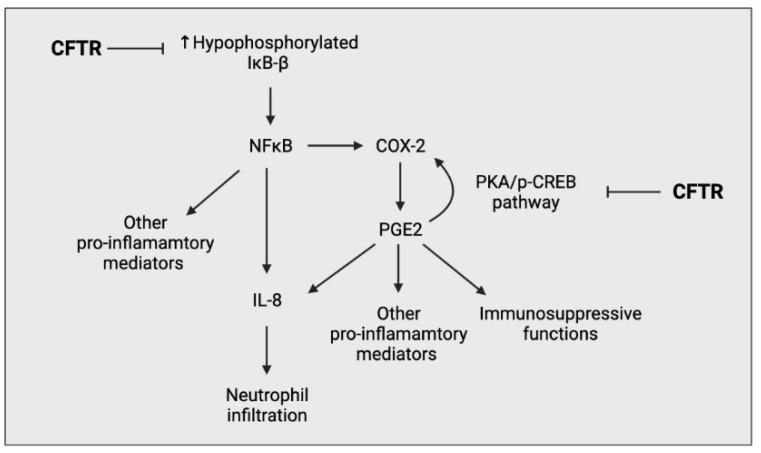
Simplified schematic outlining the ability of CFTR to negatively regulate the pro-inflammatory response of airway epithelial cells. CFTR prevents the overproduction of hypophosphorylated IκΒ-β, preventing the overactivation of the NFκΒ pathway. CFTR also negatively regulates the PGE_2_-mediated cyclooxygenase-2 (COX-2) positive feedback loop, preventing the excessive production of prostaglandin (PG) E2.

Airway epithelial cells (AECs) are a fundamental component in the progression of CF lung pathology through the secretion of pro-inflammatory mediators in response to pathogens [19]. They act as an interface between pathogens and the immune system, thus intrinsic changes induced by CFTR deficiency not only affect AECs interaction with pathogens, but also alter its interaction with other immune cells [20]. Many groups have investigated the intrinsic changes of CFTR-deficient AECs and have observed an upregulation of multiple proinflammatory processes, in particular, increased NFκB signalling and a distorted production of prostaglandins (PGs) and glutathione (GSH) [21,22,23] (Figure 4). 

Extracellular GSH is a key antioxidant within the lung epithelial lining fluid (ELF), working to scavenge free radicals and hypochlorous acid produced by neutrophils, thus protecting the lung epithelium against oxidative damage. It is reported that the ELF of CF patients contain considerably lower concentrations of GSH promoting increased susceptibility to chronic infections [24]. Gao et al. suggest that differences in GSH concentration at the apical surface of CFTR-deficient and -sufficient cells is due, predominantly, to changes in GSH efflux, indicating a direct link between CFTR function and dysfunction and GSH [22]. GSH transport was also found to be aberrated by changes in membrane potential within rat hepatocytes and isolated kidney membranes [25,26]. This may also be the case for GSH within the lung, providing an alternative, indirect relationship between CFTR function and GSH levels.

#### 2.2.2. Neutrophils

**Figure 5:**
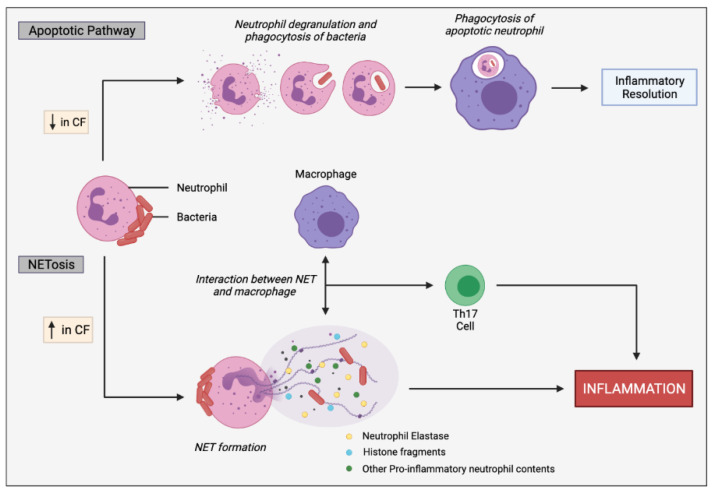
Simplified apoptotic and NETosis pathways in neutrophils. Normal apoptotic pathway involves neutrophil degranulation and phagocytosis of pathogens, initiating neutrophil apoptosis. The apoptotic neutrophil is then phagocytosed by macrophages promoting inflammatory resolution. CF neutrophils are biased towards undergoing NETosis in response to bacteria and other inflammatory stimuli, rather than the apoptotic pathway. In response to infectious stimuli, CF neutrophils expel NETs containing DNA, histone fragments and various pro-inflammatory neutrophil contents, such as neutrophil elastases which can directly or indirectly, through cell interactions, induce inflammation. Macrophages interact with the NETs, resulting in stimulation of Th17 cells, leading to neutrophil influx and further inflammation. Progressive inflammation triggered by sustained NET formation and release of toxic contents can damage the lung architecture.

Neutrophils play an irreplaceable role in maintaining efficient host defences against pathogens; however, dysregulation of neutrophil infiltration and activation can lead to immunopathy [18]. CF is characterised by an overexuberant influx of neutrophils into the airway lumen, predominantly driven by IL-17 and IL-8 secretion [27]. Excessive neutrophil infiltration and activation creates a destructive, hyper-inflamed airway microenvironment resulting in bronchiectasis and deterioration in lung function contributing to disease mortality [18]. In normal homeostatic conditions neutrophils are short-lived and undergo spontaneous apoptosis, a fundamental characteristic for encouraging the resolution of infectious and inflammatory insults [28]. The pathogenic mechanisms are believed to be heavily influenced by the secretion of neutrophil elastases (NE) and pro-inflammatory cytokines and through the formation of NETs [29] (Figure 5). 

NEs are proteolytic enzymes predominantly liberated from neutrophils following neutrophil activation into the extracellular space. Both free and membrane-bound forms of NE possess bactericidal functions at the site of degranulation and more distant regions of the body [30]. Anti-proteinases regulate NE activity at distant sites, restraining proteolytic activity to the site of infection. Surface-bound NEs, however, are resistant to endogenous inhibition, hence its activity is preserved [31]. Elevated surface-bound NE activity is suspected to drive CF lung pathogenesis by promoting bronchiectasis through lung hyperinflammation and impaired defence against pathogens, including *P. aeruginosa* [30]. Both free and membrane-bound NEs are a predictor of progressive bronchiectasis and appear to be inversely related to forced expiratory volume in one second [30]. Furthermore, increased surface-bound NE activity also correlated with reduced functional residual capacity [30]. 

NETosis involves the emanation of a mesh-like network containing DNA, histones, pro-inflammatory mediators, and other contents following neutrophil stimulation with IL-8, lipopolysaccharide (LPS) or bacteria [32]. This further aggravates airway inflammation through bacterial trapping and increased interactions with other inflammatory cells. Although the antimicrobial activity of NETosis has been reported to aid bacterial clearance during early stages of disease, its proinflammatory effects may become more detrimental rather than beneficial during chronic infections [18]. Additionally, pathogens are capable of evading NET-mediated killing by either inhibiting NET formation, promoting NET degradation or simply by developing resistance to antimicrobial constituents of NET [33]. The CF lung is highly susceptible to chronic infection of *P. aeruginosa*, which has a high tendency of developing resistance to NET-mediated killing [18,32]. 

**Figure 6:**
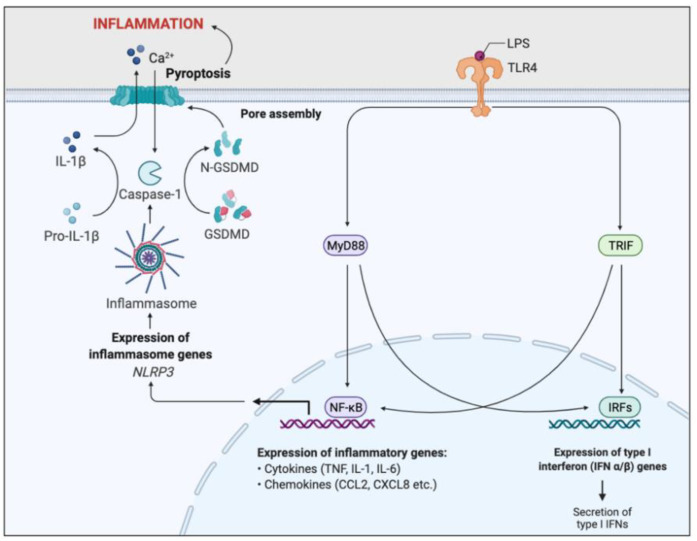
Simplified schematic of LPS-induced inflammatory pathway. Toll-like receptor 4 (TLR4) recognises and binds lipopolysaccharide (LPS). Binding activates two signal transduction pathways through the stimulation of myeloid differentiation primary response gene 88 (MyD88) and TIR domain-containing adaptor inducing IFN-β (TRIF) adaptor molecules. MyD88 and TRIF activates two transcription factors, nuclear factor-κB (NFκB) and interferon response factors (IRFs). IRFs enhance the expression of Type I interferon genes, resulting in the secretion of type I interferons. NFκB promotes transcription of pro-inflammatory genes, such as cytokines and chemokines, resulting in acute inflammation and initiation of the adaptive immunity. NFκB signalling also induces NOD-, LRR-, and pyrin domain–containing protein 3 (NLRP3) gene expression, essential for inflammasome formation. The NLRP3-inflammasome cleaves and activates caspase-1, which cleaves the inactive precursor pro-IL-1β into its biologically active and secreted form, IL-1β. IL-1β secretion results in enhanced inflammation and immune cell infiltration. Caspase-1 also cleaves and activates Gasdermin D (GSDMD), leading to pore formation in the plasma membrane, triggering pyroptosis and further inflammation. This inflammatory pathway is a key regulator of the inflammation associated with CF.

The neutrophil inflammasome and the subsequent innate immune cascade which follows is important in inflammation triggered by bacterial LPS. LPS is the primary outer-membrane component of gram-negative bacteria, in particular *P. aeruginosa*, typically present in CF [34]. It binds to pattern recognition receptors, specifically toll-like receptor 4 (TLR4), which activates the MyD88 signal transduction pathway, responsible for NFκB activation [35]. NFκB is a transcription factor which enhances transcription of pro-inflammatory genes such as cytokines and chemokines, as well as promoting NLRP3 gene expression (Figure 6).

Inhibition of the NLRP3 inflammasome formation has been found to reduce IL-1β secretion in CF airways, abrogate airway inflammation and improve the clearance of *P. aeruginosa* [36]. Inhibiting aerobic glycolysis in CF neutrophils and administering IL-1 receptor antagonists such as anakinra may also provide therapeutic benefits in ameliorating IL-1β-driven airway inflammation [36]. 

#### 2.2.3. Macrophages

Alveolar macrophages are the primary phagocytes within the airways and play a fundamental role in the innate immune system, driving the clearance of bacteria, and stimulating the adaptive immune response through antigen presentation. Following exposure to infectious stimuli, macrophages secrete a wide array of proinflammatory mediators such as IL-1, IL-6, IL-8, TNFα, and arachidonic acid metabolites, such as PGs [37]. CF macrophages possess a proinflammatory phenotype which may be associated with several factors’ characteristic of CF, such as persistent bacterial infections, changes in airway microenvironment or even from the CFTR defect itself [38,39,40]. Zaman et al. demonstrated that the MAP/Erk pathway within CF monocytes is hypersensitive to LPS stimulation, leading to increased IL-8 secretion, promoting neutrophil recruitment and a proinflammatory response against gram-negative bacteria [41]. Insufficient turnover and upregulation of TLR4 in CF monocytes may account for the hypersensitive response to LPS [42]. Perturbed TLR4 expression may also facilitate the activation of other proinflammatory pathways, such as NF-kB, MAPK, and IRF-3 pathways, inducing further inflammation and immune cell recruitment [42]. In addition to promoting an inherent proinflammatory phenotype, CFTR-deficiency has also been found to implicate the microbicidal function of CF macrophages. Zhang et al. utilised CRIPSR-Cas9-mediated CFTR KO macrophages to confirm that CF macrophage dysfunction is CFTR-dependent rather than a consequence of the CF inflammatory milieu [43]. CFTR-deficient macrophages possessed a defective microbicidal function though the inability to kill phagocytosed bacteria due to insufficient acidification of the late phagosome to a pH less than 5 [44]. An additional study observed that the Cl^-^ concentration within these phagosomes may alter the behaviour of intracellular bacteria through changes in its protein activity or host factors [45]. This alteration may modulate the evasion of bacterial killing by macrophages. Macrophages also play a key role in regulating iron metabolism within the airway microenironment, influencing the progression of inflammation and infection [46]. CF macrophages, in addition to being intrinsically hyperinflammatory, possess altered expression of iron metabolism proteins such as heme oxygenase-1, hindering its bactericidal function, as well as further promoting hyperinflammation [47,48]. Furthermore, excessive iron levels provide a nutrient-rich microenvironment for bacteria namely *P. aeruginosa*, facilitating antibiotic resistance and biofilm formation, resulting in persistent infections [49]. Studies conducted by Hazlett et al. demonstrate that the defects in iron metabolism in CF patients may be corrected upon ivacaftor and lumacaftor treatment [47].

#### 2.2.4. Lymphocytes

CF T cells appear to be skewed towards a Th17 and Th2 phenotype, rather than a Th1 dominate response, resulting in impaired antigen presentation, excessive neutrophil infiltration, and enhanced production of immunoglobulin (Ig)E [50]. 

The IL-23/IL-17 proinflammatory axis is thought to play a prominent role in driving airway inflammation in CF patients, especially those chronically infected with *P. aeruginosa* [51]. IL-23 stimulates T-cell differentiation into IL-17 secreting Th17 cells. IL-17 acts on various cell types and induces the secretion of multiple cytokines, such as TNFα, IL-6, IL-1β, and granulocyte-macrophage colony-stimulating factor (GM-CSF), triggering a robust inflammatory response [52]. This inflammatory response, whilst essential for the eradication of most bacteria, is ineffective in driving the clearance of others, most problematically *P. aeruginosa* [51]. 

The predilection to mount a Th2-mediated response in CF T cells may be the cause of the unresponsiveness to bacteria including *P. aeruginosa*, leading to increased secretion of IL-10 and lower levels of IFNγ, both of which are detrimental in neutralising bacteria [53,54]. IL-10 hinders the expression of costimulatory molecules on macrophages, preventing adequate antigen presentation required for sufficient clearance of pathogens [55]. A possible explanation for the Th2 skew is that CFTR dysfunction directly increases calcium ion Ca^2+^ influx into T cells, disturbing the gene expression critical for Th1/Th2 differentiation [56].

## 3. CFTR Modulators and Inflammation

As CF is a complex multisystem disease, efficient treatment requires a multidisciplinary and personalised approach due to the high degree of heterogeneity of CFTR dysfunction. Despite other co-morbidities, CF lung disease remains the leading cause of morbidity and mortality [5,57,58,59]. Standard treatment regimens include incorporating airway clearance techniques, such as mucolytic and hydrator therapies, in addition to regular exercise into the individual’s lifestyle from an early age [13]. Although these interventions have been a cornerstone of CF therapy, they do not target the underlying cause of disease. Recent advances in precision medicine have led to the development of CFTR modulator agents that directly target the dysfunctional CFTR.

### 3.1. Ivacaftor

**Figure 7:**
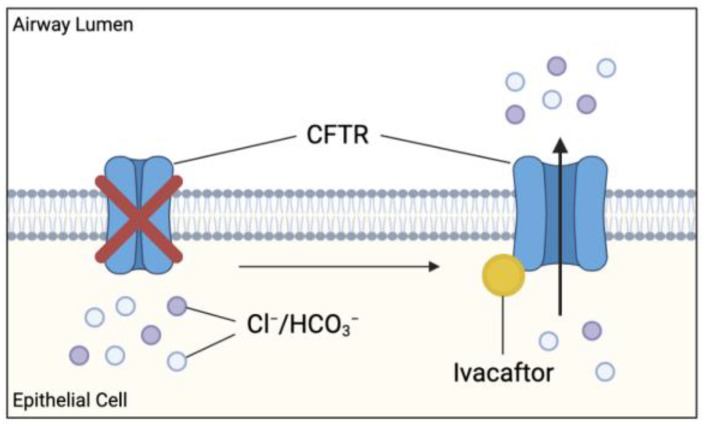
Effect of cystic fibrosis transmembrane conductance regulator (CFTR) potentiator ivacaftor on dysfunctional CFTR. Ivacaftor binds directly to CFTR protein, increasing the time which activated CFTR channel remains open at the apical membranes. Results in increased chloride and bicarbonate ion transport at these sites, possibly improving the clinical outcome in CF patients.

Ivacaftor is a small molecule potentiator originally developed to treat patients with a G551D-CFTR mutation [1]. G551D is a missense mutation affecting approximately 4% of the global CF population and causes impaired CFTR channel gating [60]. Ivacaftor monotherapy approval was later extended to include additional gating mutations, including G1244E, G1349D, G178R, G551S, S1252N, S1255P, S549N, S549R, and R117H [7]. Ivacaftor (VX-770) targets the dysfunctional CFTR and increases the time in which these channels remain open, allowing for sufficient anion transport (Figure 7). 

Theoretically, this relieves the intrinsic defect in ion transport observed in CF contributing to the hypersensitive innate immune response resulting in chronic inflammation. In particular, the resolution of chloride and bicarbonate ion transport restores the microbicidal ability of macrophages in addition to preventing the excessive Ca^2+^ influx into T cells, averting the Th2 bias. Treatment with ivacaftor is shown to drastically improve respiratory function, nutritional status, sweat chloride concentrations, and reduce the incidence of pulmonary exacerbations in these patients [61]. Since its discovery, ivacaftor has proven to be effective in almost 40 other variants of gating and residual function mutations, as well as being used in combination with other CFTR modulator drugs, extending the pool of potential recipients [1]. Clinical trials have demonstrated a positive relationship between ivacaftor treatment and clinical improvements; however, the effect on inflammation remains unclear due to conflicting results in major studies [62]. 

The G551D Observation-AL (GOAL) study investigated the effects of ivacaftor on patients aged six and above with at least one G551D allele and no prior exposure to ivacaftor [62]. They evaluated a number of clinical parameters including sputum inflammation and pathogen colonisation at 1, 3, and 6 months after ivacaftor initiation. Despite significant improvements in forced expiratory volume (FEV1) and a significant reduction in sweat chloride levels observed at 6 months compared to baseline, no significant changes in sputum inflammatory markers, nor changes in bacterial load were detected. This led researchers to speculate whether infection and inflammation could become independent of CFTR function. In contrast, a prospective study by Hisert et al. in 12 CF patients with chronic airway infections and at least one G551D-CFTR allele showed that restoring CFTR function improved patients’ lung function and sweat chloride levels consistent with the GOAL study; but reduced airway microbiota and inflammatory markers [63]. There was a rapid and significant decline in *P. aeruginosa* sputum density from day 2 of ivacaftor treatment. Furthermore, concentrations of sputum inflammatory markers, namely NE, IL-8, and IL-1β were significantly reduced within the first treatment week and continued to decline throughout the duration of the study (up to 600 days) despite the re-emergence of infection [63]. 

Additionally, ivacaftor was also found to dampen the sensitivity of peripheral blood monocytes to IFN-γ in CF patients, resulting in decreased IFN-γ-mediated responses [64]. These findings demonstrated a reduction in bacterial burden and inflammation in CF-airways [64]. Barnaby et al. found that ivacaftor alone, or in combination with a lumacaftor, markedly reduced the secretion of pro-inflammatory cytokines, such as IL-6, IL-8, TNFα, and IFNγ from *P. aeruginosa*-stimulated CF monocyte-derived macrophages [65]. Furthermore, ivacaftor has been shown to have a potential influence on neutrophil recruitment and function [66]. In brief, CF-neutrophils possess impaired degranulation of secondary and tertiary granules and defective Rab27a, responsible for granule trafficking [67]. Reduced degranulation hinders bacterial killing, however, treatment with ivacaftor was shown to restore neutrophil degranulation, significantly improving antimicrobial activity against *P. aeruginosa* [67]. In addition, ivacaftor has also been shown to significantly decrease neutrophil survival, counteracting the pro-survival phenotype present in CF [32]. By reducing NET-mediated killing, treatment with ivacaftor restores apoptosis, subsequently diminishing the inflammatory cascade triggered by NETs [32]. Despite these studies, the effects of ivacaftor on infection and inflammation in a CF lung remain uncertain. 

### 3.2. Ivacaftor Combinations

Approximately 90% of patients with CF harbour the F508del-CFTR mutation on at least one allele, and about 50% on both alleles which results in little functional CFTR reaching the cell surface [7]. Ivacaftor combinations, e.g., an ivacaftor with either lumacaftor, tezacaftor, or tezacaftor-elexacaftor, are used to target these folding mutations. Ivacaftor-lumacaftor led to modest improvements in lung function and a reduction in exacerbations in patients that are homozygous for the F508del mutation; it was ineffective in heterozygous patients [68,69]. Interestingly, macrophage phagocytosis which is responsible for the CF abnormal hyperinflammation was only restored in patients receiving ivacaftor monotherapy and not in patients receiving ivacaftor-lumacaftor therapy [70]. As lumacaftor, a cytochrome 3A4 inducer, has been shown to interfere with ivacaftor concentrations (a cytochrome 3A4 substrate), it is likely that the drug interactions that occur between lumacaftor and ivacaftor interfere with the ability of ivacaftor to stimulate bacterial phagocytosis [71,72,73]. In line with these findings, whole blood transcriptomics showed that patients homozygous for the F508del-CFTR mutation displayed substantial and continuous overexpression of a myriad of inflammatory genes which persisted unchanged under ivacaftor-lumacaftor therapy [74]. Interestingly, an in vitro study conducted by Gentzsch et al. showed the opposite, namely that lumacaftor-induced correction of the defective F508del-CFTR improved the inflammatory environment of the CF airway [75,76]. Kopp et al. studied whole-blood transcriptomic responses to lumacaftor/ivacaftor therapy in CF [74]. They found that pre- and post-drug CF profiles were associated with marked overexpression of inflammation-related genes and apoptosis genes, and significant under-expression of T cell and NK cell-related genes compared to healthy subjects. Ivacaftor-lumacaftor responders demonstrated changes in eIF2 signalling, oxidative phosphorylation, IL-17 signalling, and mitochondrial function compared to non-responders.

Ivacaftor-tezacaftor was shown to be efficacious in F508del heterozygous patients carrying a CFTR residual function allele, as well as displaying comparable clinical efficacy outcomes, e.g., FEV_1_ [69,77]. The triple ivacaftor-tezacaftor-elexacaftor combination is approved for all CF patients 12 years or older carrying the F508del mutation or one of 177 other approved mutations. Importantly, in patients homozygous for F508del-CFTR, the addition of elexacaftor has decreased pulmonary exacerbations by 63% compared to ivacaftor-tezacaftor treatment which showed a reduction by 35% compared to placebo [75]. As the tezacaftor containing combinations have only recently been approved, studies to date are still limited; however, it is likely that the different CFTR modulator combinations (down)regulate inflammation differently.

## 4. Conclusions

Chronic lung inflammation is a hallmark of CF and contributes to progressive respiratory decline. The extensive influx of pro-inflammatory cells and mediators into the CF lung drives the pernicious cycle of inflammation leading to lung tissue damage and increased susceptibility to microbial infections. Due to the intricate nature of inflammation, it is challenging to elucidate the pathways leading to immunopathy; and the key inflammatory mediators and immune response involved in CF have yet to be fully elucidated. The recent development of CFTR modulator drugs, namely ivacaftor, has reinvigorated the CF community as it directly resolves the CFTR dysfunction, relieving numerous pulmonary and extra-pulmonary clinical manifestations of the disease. Many researchers have suggested that ivacaftor may reduce lung inflammation in CF patients, however, results from current literature remain inconclusive [78]. Recent in vivo data revealed a positive correlation between airway inflammation and CFTR modulator-induced improvement in lung function, suggesting that inflammation is a key regulator of HCO_3_^−^ secretion in CF airways. However, further research is required to distinguish whether a prophylactic or therapeutic administration of ivacaftor combats inflammation. Further knowledge of the exaggerated inflammatory response within the lungs of CF patients remains a pillar for understanding the disease as a whole and is integral for the development of more efficient and personalised therapeutic regimens to improve not only the survival, but the quality of life of each individual with CF. 

## Data Availability

Not applicable.

## Abbreviations

AEC, airway epithelial cell; ASL, airway surface liquid; BALF, bronchoalveolar fluid; CF, cystic fibrosis; CFTR, Cystic fibrosis transmembrane conductor regulator; COX-2, Cyclooxygenase-2; ELF, Epithelial lung lining fluid; G551D, Gly551Asp; GM-CSF, Granulocyte-macrophage colony-stimulating factor; GSH, Glutathione; IFN, Interferon; Ig, Immunoglobulin; IL, Interleukin; LPS, Lipopolysaccharide; NE, Neutrophil elastase; NET, Neutrophil extracellular traps; NETosis, NET-mediated cell death; NFκB, Nuclear factor-κB; NLRP3, NOD-, LRR-, and pyrin domain–containing protein 3; PG, Prostaglandin; Th, T helper; TLR4, Toll-like receptor 4; TNF, Tumour necrosis factor.
